# Urological recommedations of Hadji Pasha’s, a Turkish aged doctor in Anatolia

**Published:** 2016

**Authors:** Mehmet Erol Yıldırım, Metin Canbal, Ekrem Ozyuvali, Ömer Faruk Karataş

**Affiliations:** 1 *Department of Urology, Turgut Özal University School of Medicine, Ankara, Turkey*; 2 *Department of Family Medicine, Turgut Özal University School of Medicine, Ankara, Turkey*; 3 *Department of Urology, Atatürk Research and Training Hospital, Ankara, Turkey*

**Keywords:** *Hadji Pasha*, *Urology*, *Andrology*, *Phytotheraphy*

## Abstract

**Objective:**

Urinary tract conditions have been an important part of diseases from antiquity until today. Historically, many plants and herbs have been used for the treatment of urinary disorders.

**Methods:**

Celâlüddîn Hızır bin Ali el-Konevi (Hadji Pasha) is one of the most famous physician who lived in Anatolia between 13th and 14th centuries. He has written one of the most important medical books of that era, "Müntehab-ıŞifa" (solution of wellness) in Turkish. General medical information about the diseases in this book, focus on diagnosis and treatment.

**Results:**

The herbal solutions for urological disorders such as, urinary incontinence, urinary stones or erection problems are told in this section.

**Conclusion:**

Many of the herbal medicines addressed in this book are being widely used in current medicine, but the usage of these herbals in daily urology practice is limited. In this study, we aimed to share the advices for the urological diseases and the related herbal medicines that are named in Hadji Pasha’s book, "Müntehab-ıŞifa ", with today's physicians.

## Introduction

Doctor Hadji Pasha’s real name was Celâlüddîn Hızır and his date of birth is not precisely known but was approximately 1335 AD. He went to Cairo, Egypt for theological education after he graduated from his Madrasa education in Konya, Turkey. After becoming severely ill in Cairo, he developed a great interest in the medical sciences. Upon successful completion of his medical education, he became a physician and chief of staff in Mansuriye Kalavun Hospital in Cairo. The encouragement of Isa Bey who helped him during his education influenced him in his medical profession (Ustun, 2010 ▶). 

When physician Hadji Pasha went to Birgi and Ayasuluk (Seljuq-Selçuk, Izmir) at the invitation of Aydinoglu Isa Bey (1380 AD), he continued his scientific and medical studies under the protection and support of the sultanate. It is a fact that Hadji Pasha, who worked as a Hodja (teacher) at the Madrasas (college) of Birgi and as a physician at the palace besides being an adjudicator of Ayasuluk, was greatly respected as a Turkish physician by the monarch and palace’s high officials. He published the Şifâul- Eskâm (treatment of illnesses) and Deva-ül- Alâm” (treatment of symptoms) in Ayasuluk under the name of İsa Bey. He acquired the title Ibn-iSina (Avicenna) of Anatolia through this book, which was also called “the Canon” of Hadji Pasha. In his medical studies, he took Galenos and Ibn-iSina as models. The date of his death is not precisely known and estimated to be 1424 AD. His grave is in the Hidirlik area in Birgi/Odemis.

 It is rumored that the name Hadji Pasha was given to him by the Aydinogullari after he returned from Cairo (Ustun, 2010 ▶; Batuta, 1983 ▶). He had many books about theology, philosophy and medicine named Kitab’ul Teâlîm (book of education), Kitab’ul-Feride (book of rare diseases), Sifâ’ul-Eskâm and Devâul–Alâm, El-Usûlul-Hamse (methods of five) and Müntehab-ıŞifa (solution of wellness). He wrote about all fields of medicine in his book “Müntehab-ıŞifa”, from which we will give some recommendations about urological disorders. 

## Material and Methods

Here we are presenting the definitions and recommendations of Hadji Pasha on some urological disorders based on “Müntehab-ıŞifa” which are currently being used. First, the manuscript was transliterated into contemporary Turkish a few years ago. It has not been translated to another language yet. We tried to give the names of the herbals also in Latin, in order to be understood by all scientists. Since we could not find some of the herbals names in daily Turkish, we did not translate them to English and Latin. Also, we searched PubMed in order to find the current urological usage of the herbals that are written in the book. 

## Results

Difficulty in urination must have been due to the urinary tract infection (UTI), weakness of bladder or obstruction. After boiling of fennel (*Foeniculum vulgare*) and violet (*Viola tricolor*), patient must drink it with honey. 

In bladder stones, if the urination is difficult and urine is full of sediments, it is the sign of bladder stone. If the patient takes the mixture of melted plum (*Prunus domestica*) and 30 g of celery seed (*Apium graveolens*) for ten days, it will crash the stone and helps to expelit. To melt down the bladder stone, boiled blackberries also should be taken.

For ureteral and kidney stones,if the patient drinks the mixture of crushed blueberries (*Vaccinium myrtillus*) and cinnamon (*Cinnamomum zeylaniccum*) 6g with milk, it will resolve the stones. Also, crushed egg shell was added to pomegranate (*Punica granatum*) will resolve the urinary stones. In addition to these, Coven grass (*Gypsophila*) may help to expel the urinary stone.

Urinary incontinence may be due to UTI and cold weather. Forty nodes (*Cyperus longus*), lavender herb (*Lavandulastoechas*), cumin (*Thymus serpyllum*), fennel (*Foeniculum vulgare*), daily grass (*Gummi olibanum*), buckthorn (*Cyprus rotundum*) should be taken either pasted or mixed with sugar three times a day. Patient must be aware of sour, salty and solid foods. Eating of dried wheat after 3 days of wetting period will be useful in a few days for urinary incontinence.

Urinary tract infection, dysuria, sharp smell of the urine, urethral discharge and cloudy urine indicate the presence of lesionsin the bladder and kidney. Contemporary patient should eat noodles (Lenticula), spinach (*Spinacia oleracea*), pumpkin (*Cucurbita pepo*), purslane (*Portulaca oleracea*), almond oil (*Amara Amygdalinas oleum*), stinging nettle (*Urtica dioica*) and boiled eggs. 

For the treatment of enuresis nocturna, one should take fried sweet basil (*Ocimum basilicum*) 4 g a day or adding 4 g of burned silk to the children's’ food only once may stop nocturnal enuresis. 

To increase the libido,men should eat goose meat, hazelnuts (*Corylus avellana*), figs (*Ficus carica*), onions, black pepper (*Piper nigrum*), curcumin (*Zingiber officinale*), stinging nettle (*Urtica*), chickpea (*Cicer arietinum*), mustard (*Sinapis arvensis*), melon seeds (*Cucumis melo*), boiled egg, milk, yoghurt, grapes (*Ribes rubrum*), turnips (*Brassica napobrassica*), radishes (*Raphanus sativus*), peanut (*Pistacia vera*), coconut (*Cocos nucifera*), almonds (*Amygdalus communis*) , orchid (*Orchis maculata*). 

To decrease libido, men should take pearl grass (*Ruta graveolens*), bean seed (*Lupinus albus*), flaxseed (*Linum usitatissimum*), cumin (*Cuminum cyminum*), hartnup seed (Ceratonia siliqua).

To increase erection, men should take hazelnuts (*Corylusavellana*), Fructuscubebae.

To increase the semen volume, men should take saffron (*Crocus sativus*), fresh dates (*Phoenix dactylifera*), stinging nettle (*Urtica dioica*).

To decrease the semen volume, men should take Ruta graveolens, garlic (*Allium sativum*). 

## Discussion

According to the decisions of the World Health Organization (WHO), developing countries were encouraged to utilize traditional medicine in situations that modern medicine approach is not effective (Pal et al., 2003 ▶). Thus, currently, the world witnesses a rising propensity towards herbal remedies. Studies stated that over 80% of people in developing countries apply herbal remedies for curative needs (Mikaili et al., 2011 ▶). Recently, the employment of complementary medicine in patients with urinary disorders is increasing. However many of the herbals that were discussed in this paper are widely used in medicine, their current usage in urology is limited. 

There is a rat study hypothesizing the reducing effect of *Prunus domestica* on BPH (Swaroop et al., 2015 ▶).* Apium graveolens* has been shown to increase the spermatogenesis in rats (Hardani et al., 2015 ▶). We have shown the antioxidant effect of *Zingiber officinale* on ischemia-reperfusion damage in rat kidneys (Uz et al., 2009 ▶). There are few studies that have shown anti-hyperplasic effect of *Urtica* in rats (Moradi et al., 2015 ▶)*.*

## Conclusion

In the present study, it was found that the principles of detecting urinary tract illnesses are highly similar. Also, some of the recommended herbal treatments are being widely used nowadays. 

## Note

*Please cite this paper as: *

Yıldırım ME, Canbal M, Ozyuvali E, Karataş OF. Urological recommedations of Hadji Pasha’s, a Turkish aged doctor in Anatolia.Avicenna J Phytomed, 2016; 6 (5): 502-505.
